# Positive fortune telling enhances men’s financial risk taking

**DOI:** 10.1371/journal.pone.0273233

**Published:** 2022-09-07

**Authors:** Xiaoyue Tan, Jan-Willem van Prooijen, Paul A. M. van Lange

**Affiliations:** 1 Erasmus School of Philosophy, Erasmus University Rotterdam, Rotterdam, South Holland, The Netherlands; 2 Department of Experimental and Applied Psychology, VU Amsterdam, Amsterdam, North Holland, The Netherlands; 3 The Netherlands Institute for the Study of Crime and Law Enforcement (NSCR), Amsterdam, The Netherlands; 4 Department of Criminal Law and Criminology, Maastricht University, Amsterdam, The Netherlands; Universidad de Murcia, SPAIN

## Abstract

Fortune telling is a widespread phenomenon, yet little is known about the extent to which people are affected by it—including those who consider themselves non-believers. The present research has investigated the power of a positive fortune telling outcome (vs. neutral vs. negative) on people’s financial risk taking. In two online experiments (*n*1 = 252; *n*2 = 441), we consistently found that positive fortune telling enhanced financial risk taking particularly among men. Additionally, we used a real online gambling game in a lab setting (*n*3 = 193) and found that positive fortune telling enhanced the likelihood that college students gambled for money. Furthermore, a meta-analysis of these three studies demonstrated that the effect of positive fortune telling versus neutral fortune telling was significant for men, but virtually absent for women. Thus, positive fortune telling can yield increased financial risk taking in men, but not (or less so) in women.

## Introduction

“*Men become superstitious*, *not because they have too much imagination*, *but because they are not aware that they have any*.”

—George Santayana, Atoms of Thought

Superstition is defined as a subset of paranormal beliefs that are used to bring about good luck or avoid bad luck [1]. For example, it is superstitious to believe that knocking on wood can prevent bad luck, because luck depends on chance and there is no rational reason to assume any causal influence of touching wood. Believing superstition and engaging in superstitious activities is pervasive throughout history and across cultures [1, 2], and is still prevalent in our modern society [3]. More than half of Americans reported that they performed some kind of superstition that they believed in [4]. Some research indicates that people are willing to pay more for products (e.g., license plate, house, and telephone number) that contain lucky numbers [5–7], while they are less willing to do business on Friday the thirteenth [8]. The ubiquity of superstition in human beliefs and thinking is also underlined by the large number of newspapers and magazines that regularly publish horoscopes, and a plethora of websites that offer online fortune telling services.

In spite of the popularity of relying on superstition in people’s life, scientific understanding of superstition is still rather limited. Because of its irrational or paranormal nature, believing in superstition is often considered as nonscientific and unfounded [9], or as a failure of human rationality [10, 11]. Recently researchers started to explore and recognize the psychological benefits of believing in superstition, however, particularly in adverse situations. People have a strong need for recognizing causal (over noncausal) relationships regardless of whether these relationships are true [12]. Hence, it is common for many people, including intelligent and mentally stable adults, to be susceptible to superstition. This holds even for many people who claim to not believe in it [13]. Research suggested that people rely on superstition to combat feelings of uncertainty, high levels of psychological tension, and low levels of perceived control [14–17]. Furthermore, performing superstitious rituals can be a functional coping mechanism after losses [18], and may improve performance by boosting people’s confidence and increasing their task persistence [19].

The present research is grounded in the assumption that, although people may not readily admit to it, superstition can play an important role in risky decision-making. In our studies we examine the effects of one intriguing expression of superstition–fortune-telling–that has received virtually no empirical attention in past research. Moreover, we examine the effects of fortune telling on people’s subsequent financial risk-taking, a domain that is clearly involving and consequential in life. In three experiments, we therefore test whether people take more financial risks following a positive fortune-telling outcome as opposed to a negative or neutral one, even among people who indicate to not believe in fortune telling.

### Fortune telling and financial decision-making

It is uncommon for human beings to be perfectly rational when making decisions in complex situations [20]. Nevertheless, people can be calculative and weigh the benefits and drawbacks of different decision-making options and estimate the likelihood of success. Often the consequences of decisions are unpredictable, which depend fully or partially on chance. This also applies to financial decision making, which concerns the attainment, employment, allocation, and distribution of resources. Factors such as uncertainty, ambiguity, and risk, often are unavoidable during people’s financial decision making. In such situations, superstition may influence such decisions by adding a sense of predictability to the outcomes of one’s choices.

Superstition can be a major strategy for businessmen in coping with uncertainty in some cultures [9]. For example, it is common that Chinese businessmen consult *feng shui* experts for advice when they feel threatened by uncertain or unknown situations during strategic decision-making processes. A study [21] revealed that numerological superstition affected financial decision-making in the Chinese initial public offering (IPO) market. Lucky numerical stocks are offered with a higher frequency on the China initial public offering. Although these studies are exploratory in nature, the empirical evidence from these studies suggests associations between superstition and financial decision-making.

Fortune telling is one of the most popular forms of superstition [22]. Fortune telling is defined as the practice of predicting information about a person’s life, using for example horary astrology, cartomancy or crystallomancy [23]. People often consult fortune tellers, *feng shui* experts for instance, or fortune telling websites for their services. People who believe fortune telling hold superstitious beliefs that their future fortune is predetermined, and that their future can be predicted by fortune tellers based on information about basic external factors such as their birthdates [24]. In spite of its popularity in people’s daily life, there are almost no studies investigating the effects of fortune telling on people’s subsequent behaviors. We could only find a few studies that examined the influence of luck on consumer judgement and behavior. For example, [25] found that priming Chinese participants with luck-related stimuli (e.g., number “8” versus number “4”; the former is believed to bring good luck in Chinese culture, while the latter is believed to bring misfortune) enhanced people’s financial risk taking. Therefore, it is likely that a randomly assigned positive fortune telling outcome, which we called ‘illusory good luck’, would enhance people’s financial risk taking as compared to a neutral or negative one. Consistent with the claim that people act upon superstition even when they claim to not believe in it [13], we predicted that these effects would emerge even among people who claim not to believe in fortune telling. With the first attempt to experimentally test the influence of fortune telling on people’s financial decision-making, we aimed to provide insights into the role of superstition in people’s financial decision-making.

The pursuit and maintenance of control has been argued to be a key human motivation [26, 27]. There is a vast empirical social science literature about how people psychologically cope with uncertain events with uncontrollable outcomes. According to [28], in these situations, people either fall in a state of learned helplessness [29] or rely on superstitious behaviors to generate an illusion of control [30]. Some researchers [27] also claim that when facing uncontrollable situations, people preserve a sense of order and non-randomness through various psychological defenses, including adhering to superstitions. Such defenses may combat anxiety and discomfort from lack of personal control. Furthermore, in the present research we explored the possible psychological effects that can be brought about by a fortune-telling outcome. Specifically, we explored whether a positive fortune-telling outcome would bring people stronger feelings of control over their future or stronger general self-efficacy. These assumptions are also based on findings that superstition brings people psychological benefits in certain situations. For example, superstitious rituals can increase people’s feelings of control after losses [18] and promote stronger confidence in an uncertain and psychologically demanding situations [16, 19]. Thus, we aimed to provide greater insight into the underlying psychological mechanisms for the proposed effect of a positive fortune-telling outcome on people’s subsequent financial risk-taking.

## Study 1: Fortune-telling and financial risk taking

In Study 1, participants were randomly assigned to fortune-telling outcome conditions (positive, negative or neutral), which allegedly were based on basic information such as birth dates, birth places and favorite colors. After a neutral distractor task, we then asked them to complete a situational judgment test on financial risk taking [31].

### Method

#### Participants

We recruited participants through the online platform CrowdFlower ($0.75 compensation), and the sample contained a total of 266 participants who completed the study. Twelve participants were excluded because at the end of the survey, they either indicated to not have answered all the questions seriously or reported that they were not able to complete the survey without any distractions in the background. In addition, two observations diagnosed as outliers (These two cases were with a standardized residual greater than 3 in the dependent measure—financial risk tolerance.) were excluded, leaving a total of 252 valid cases (46.4% female; *M*_age_ = 34.04, *SD* = 12.50; 81% at least had some college education level; 96% English native speakers).

#### Procedure and measures

The research proposal entitled "The influence of fortune telling on financial risk taking" was approved by the Scientific and Ethical Review Board (VCWE) of the Faculty of Behavior & Movement Sciences, VU University Amsterdam, on 22nd April 2016. Participants filled out informed written consent before taking part in the study and all the data were analyzed anonymously. Participants were provided with an online link to the experiment presented with Qualtrics survey software. At the beginning of the experiment, participants were asked to give their informed consent to participate in three small studies that would take 15 minutes in total. We told them that the first study would test people’s satisfaction with an easy app developed for predicting people’s future fortune based on demographic information; the second study would test people’s recognition of six common materials, including leather, glass, metal, paper, plastic and fabric; and the third study would be about financial decision-making. After participants agreed to participate, we asked them to provide some demographic information (e.g., gender, age, native language, and education level) and start with the first study.

In ‘Study one’, we introduced a fortune-telling app, which was described as a tool to predict people’s fortune based on specific information such as birth date and favorite colors. After participants had submitted this information, they received a brief prediction of their future. We manipulated the fortune-telling outcome by randomly providing participants with different predictions about their future fortune and financial states.

In the neutral condition, participants received the following outcome: *“Dear Sir or Madam*, *almost 200 people recorded in our data set share at least 90% of the most important basic information with you*. *According to the estimation from our app*, *you are a reliable*, *charismatic and interesting person*. *Sometimes*, *you can be serious*, *thoughtful and even indecisive*. *These qualities will continue to influence your life in many ways*, *both in the near and distant future*. *Many thanks for your experience*! *All your information will be kept confidential and anonymous*.*”*

In both the positive and negative fortune telling outcome conditions, the outcome was the same except that it also included a prediction of participants’ future financial state. In the positive condition, participants read “*The app predicts that you will have a lot of luck in the financial domain*. *Chances are big that you will always have enough money for a high-quality life*”, while in the negative condition, participants read “*The app predicts that you will not have much luck in the financial domain*. *Chances are small that you will always have enough money for a high-quality life*.”

After the fortune-telling outcome, we asked participants to rate their experience of using the fortune-telling app. Then, we asked participants to continue with ‘Study two’, in which we presented participants with 24 images of different materials one by one and asked them to categorize them correctly by selecting the right category among six categories, notably leather, glass, metal, paper, plastic and fabric. We told participants that each image would stay on the screen for only five seconds, and that they needed to click the correct category for each image as quickly as possible. Moreover, participants were informed that their reaction time would be recorded. ‘Study two’ was designed to distract participants’ attention from the fortune telling outcomes, to minimize any influence of demand characteristics.

After ‘Study two’, we told participants to continue with ‘Study three’, which contained our measure of financial decision-making. We used nine selected items (See the S1 File) from the financial risk tolerance assessment [31] to assess participants’ financial risk taking. One example item was “When you think of the word ’risk’ which of the following words comes to mind first? a. Loss, b. Uncertainty, c. Opportunity, d. Thrill”. Another example item was “If you unexpectedly received $20,000 to invest, what would you do? a. Deposit it in a bank account, money market account, or an insured CD, b. Invest it in safe high-quality bonds or bond mutual funds, c. Invest it in stocks or stock mutual funds”. Participants’ responses to each question were scored using the scoring table from [31] (α = 0.72).

We also asked participants what fortune-telling outcome they received, to check the effectiveness of our manipulation. At the end, we rated participants’ beliefs in fortune telling. We asked them to move the slider from the neutral position to indicate to what extent they believe in the prediction given by the fortune telling app, and in general how credible they found predictions given by fortune tellers. Here we used a 100-point scale, in which “0” means “Not at all” and “100” means “A great deal”. The correlation between these two items was high (*r* = .71, *p* < .001), and hence, we averaged them into a single index of beliefs in fortune telling. Participants were debriefed at the end of the research.

### Results

#### Manipulation checks

Most of the participants (around 80%) were able to accurately recall which fortune-telling outcome they had received in the research: 79.5% in the negative condition, 74.7% in the neutral condition, and 77.9% in the positive condition. This suggests that, in general, participants perceived the manipulation of fortune-telling outcome as intended.

#### Beliefs in fortune telling

We conducted a one-sample t-test to test the difference between participants’ reported beliefs in fortune telling (*M* = 35.66, *SD* = 28.52) and the scale mid-point of 50. Result revealed that participants’ reported beliefs in fortune telling was significantly lower than the neutral midpoint, *MD* = −14.34, *t* (251) = -7.98, *p* < .001, indicating that participants mostly did not believe in fortune telling. Interestingly, we found there was a significant gender difference in beliefs in fortune telling. Compared to women (*M* = 28.04, *SD* = 26.69), men (*M* = 42.26, *SD* = 28.50) believed in fortune telling more strongly, *MD* = 14.21, *t* (250) = 4.07, *p* < .01. Moreover, we found a significant positive relationship between participants’ beliefs in fortune telling and their financial risk tolerance level, *r* = .32, *p* < .001.

The descriptive analysis for people’s financial risk tolerance by gender among different fortune telling conditions is displayed in Table 1. Due to a significant relationship between beliefs in fortune telling and financial risk taking, we conducted an ANCOVA with beliefs in fortune telling as a covariate to test the effects of fortune telling outcomes on people’s financial risk taking. We also explored the effect of gender and the interaction effect of gender and fortune telling outcomes in this model.

**Table 1:** Descriptive statistics for financial risk tolerance by gender among different fortune telling conditions in Study 1.

	Fortune telling conditions	*N*	*Mean*	*SD*
Women	Negative	45	14.22	3.03
	Neutral	31	15.84	3.06
	Positive	41	15.37	3.21
Men	Negative	38	17.21	3.92
	Neutral	52	16.81	3.35
	Positive	45	18.71	4.34
Total	Negative	83	15.59	3.76
	Neutral	83	16.45	3.26
	Positive	86	17.12	4.17

#### Effects of fortune telling

As expected, beliefs in fortune telling had a significant effect on participants’ financial risk tolerance level, *F* (1, 245) = 21.17, *p* < .001; η_*p*_^2^ = .080. The main effect of fortune telling outcomes on participants’ financial risk tolerance was significant, *F* (2, 245) = 4.54, *p* = .012; η_*p*_^2^ = .036. When participants received a positive fortune telling outcome, they had higher financial risk tolerance than when they received a negative fortune telling outcome (*M*_negative_ = 15.59, *SD* = 3.76; *M*_neutral_ = 16.45, *SD* = 3.26; *M*_positive_ = 17.12, *SD* = 4.17). According to multiple comparisons adjusted by Bonferroni, the difference in financial tolerance level reached significance, but only between the positive and negative conditions, *MD* = 1.59, *SE* = 0.53, *p* < .01. Consistent with previous findings [32], a strong gender difference in financial risk tolerance appeared in this study, *F* (1, 245) = 18.00, *p* < .001; η_*p*_^2^ = .068. Compared to women (*M* = 15.05, *SD* = 3.15), men (*M* = 17.56, *SD* = 3.92) showed higher level of financial risk taking.

**Fig 1:**
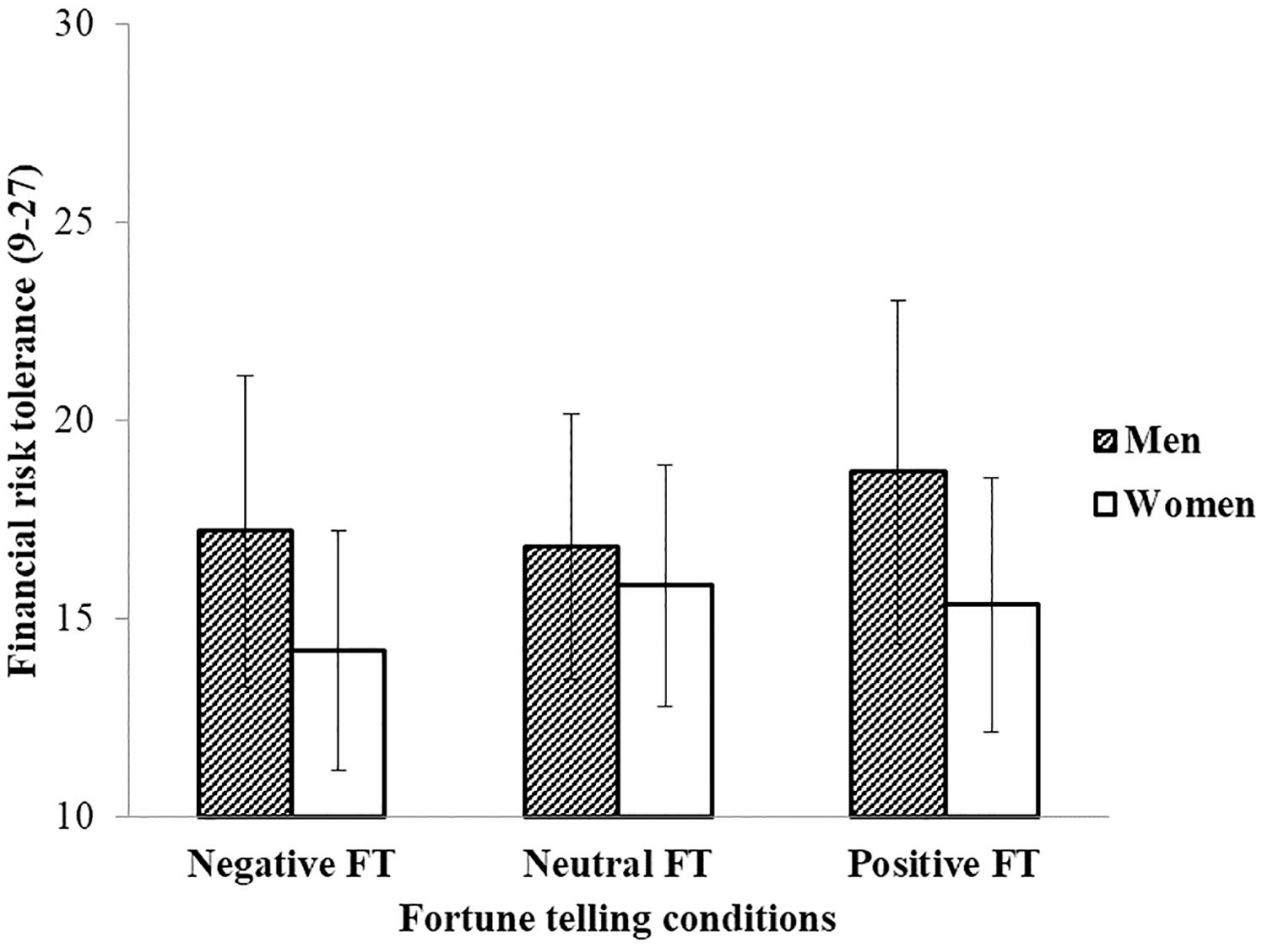
Mean financial risk tolerance among fortune telling conditions for men and women. Error bars represent standard deviations in Study 1.

Interestingly, there was a trend that gender moderated the effects of fortune telling conditions, *F* (2, 245) = 2.99, *p* = .052; η_*p*_^2^ = .024. Further regression analysis (All relevant data sets and analysis outputs, including all the whole regression tables with regression statistics are available in Zenodo: https://doi.org/10.5281/zenodo.5522672. We have also created a supplemental file: S1 File under Supporting information) indicated that men and women responded differently to a positive fortune telling outcome, *b* = − 2.43, *t* = − 2.28, *p* = .023 (For full regression results, see S1 and S2 Tables in S1 File). Specifically, for men, receiving a positive fortune telling outcome greatly enhanced their financial risk tolerance as compared to receiving a neutral one, *b* = 1.97, *SE* = 0.69, *t* = 2.86, *p* = .005, 95% confidence interval (CI) = [0.62, 3.33] (See S1 Table in S1 File). By contrast, the difference between positive and neutral fortune telling condition was not significant for women, *b* = − 0.45, *SE* = 0.81, *t* = − 0.56, *p* = .576, 95% confidence interval (CI) = [−2.03, 1.13] (See S2 Table in S1 File). In brief, receiving a positive fortune telling prediction substantially increased financial risk taking among men, whereas women were unaffected by it. The results are displayed graphically in Fig 1.

### Discussion

Study 1 revealed that although people in general did not believe in fortune telling, a positive (versus negative) fortune-telling outcome did enhance people’s financial risk taking. Furthermore, stronger beliefs in fortune telling predicted increased financial risk tolerance. It is noteworthy that men tolerated greater risk in financial decision making than women. Of importance, the risk-enhancing effect of positive fortune telling (versus neutral fortune telling) emerged only among men and not among women.

The finding that the effects of fortune telling were moderated by gender was unexpected and stimulated additional predictions for the next study. Gender differences in risk taking have been frequently reported in the literature. [33] have conducted a meta-analysis of 150 studies focusing on gender difference in risk-taking tendencies, and found that independent of age, men are more inclined to take gambling risks than women. In addition, compared to women, men are also affected more strongly by positive superstitious beliefs in lucky numbers [34]. These findings suggest that our Study 1 findings about gender may not be a statistical anomaly: Men, as compared to women, are more motivated to take risks in the financial domain, and their risk-taking responses may therefore be especially sensitive to fortune-telling outcomes. In Study 2, we tested if we could get further evidence for this proposition.

## Study 2: Fortune telling in general and risk taking across domains

Study 1 provides preliminary evidence for the idea that people are susceptible to fortune telling outcomes even when they are generally skeptical of it. We also found that gender was an important factor in these effects, as men took more financial risks after a positive fortune telling outcome, whereas women were unaffected by it. Study 2 was designed to complement Study1 in the following ways. First, we also measured other life domains than the financial domain to test if the effects found in Study 1 would generalize to other types of risk-taking behavior. Second, we provided more generally formulated fortune telling predictions rather than specific ones in the financial domain in both positive and negative fortune telling conditions. Third, we examined a possible mediator of these effects. Since superstition can boost people’s self-efficacy [19], and high perceived self-efficacy increases risk taking [35], we explored whether positive fortune telling would boost participants’ general self-efficacy, and subsequently affect their risk-taking.

### Method

#### Participants

We sought to recruit 500 participants through the online platform CrowdFlower ($ 0.75 compensation), and the sample contained a total of 477 complete responses. We excluded 36 participants because they either indicated they did not answer all the questions seriously, or they reported they were not able to complete the survey without any distractions in the background at the end of the survey, leaving a total of 441 cases (47.8% female; *M*_age_ = 34.14, *SD* = 11.25; 84.1% had at least some college education level; 90.5% English native speakers).

#### Procedure and measures

The research proposal entitled "The influence of fortune telling on financial risk taking" was approved by the Scientific and Ethical Review Board (VCWE) of the Faculty of Behavior & Movement Sciences, VU University Amsterdam, on 22nd April 2016. Participants filled out informed written consent before taking part in the study and all the data were analyzed anonymously. The procedure was almost the same as in Study 1. One major difference from Study 1 was that we used more generally formulated fortune telling predictions instead of specific fortune telling predictions in the financial domain in both positive and negative fortune telling conditions. For example, in the positive condition, we stated “*The app predicts that you will be lucky enough to have a prosperous life in the future*. *You will always have the opportunity to express your talent and can achieve remarkable success*. *Chances are big that there will always be enough money for a high-quality life*. *Meanwhile*, *you will easily acquire many good friends and have high quality close relationships throughout your life*.” In contrast, in the negative condition we stated “*The app predicts that you will not be lucky enough to have a prosperous life in the future*. *You will not always have the opportunity to express your talent and cannot achieve remarkable success*. *Chances are small that there will always be enough money for a high-quality life*. *Meanwhile*, *you will not easily acquire many good friends and will have no or troublesome close relationships throughout your life*.”

Another major difference from Study 1 was that besides the same financial risk tolerance scale that we used in Study 1, we added additional risk-taking measures. We used existing likert-type self-assessments of risk taking across different domains, including ethical, financial, health or safety, recreational and social domains [36]. This allows us to examine whether a manipulated fortune-telling outcome (positive, negative or neutral) would affect people’s risk taking across different life domains. We provided 30 statements, and for each we asked participants to indicate the likelihood that they would engage in the described activity or behavior if they were to find themselves in that situation. We asked participants to provide a rating ranging from *Extremely Unlikely* to *Extremely Likely*, using a scale from 10 to 70. Example items are: “Taking some questionable deductions on your income tax return.” (Ethical domain; six items in total; α = .87); “Betting a day’s income at the horse races.” (Financial domain; six items in total; α = .84); “Driving a car without wearing a seat belt.” (Health or safety domain; six items in total; α = .78); “Going down a ski run that is beyond your ability.” (Recreational domain; six items in total; α = .85) and “Having an affair with a married man/woman” (social domain; six items in total; α = .62).

Additionally, we included a general self-efficacy scale [37] for exploratory purposes. An example item for the self-efficacy was “I can always manage to solve difficult problems if I try hard enough.” Another example item was “I am confident that I could deal efficiently with unexpected events.” There are ten items in total for this scale (α = .82).

At the end, we rated participants’ beliefs in fortune telling in the same way as it was in Study 1. The correlation between the two items was high (*r* = .73, *p* < .001), and we again averaged participants’ responses into an index of beliefs in fortune telling.

### Results

#### Manipulation check

Most of the participants (from 72.6% to 82.8%) were able to accurately recall which fortune telling outcome they had received in the study: 77.2% in the negative condition, 82.8% in the neutral condition, and 72.6% in the positive condition. These findings again indicate that most participants perceived the manipulation of fortune telling outcome as intended.

#### Beliefs in fortune telling

Again, we conducted a one-sample t-test to examine the difference between participants’ reported beliefs in fortune telling (*M* = 36.10, *SD* = 28.83) and the neutral scale mid-point of 50. Result revealed that participants’ reported beliefs in fortune telling was significantly lower than the neutral scale midpoint, *MD* = −13.90, *t* (440) = −10.12, *p* < .001, indicating that in general, participants reported to not believe in fortune telling. Again, we found there was a significant gender difference in beliefs in fortune telling. Compared to women (*M* = 29.66, *SD* = 26.38), men (*M* = 42.01, *SD* = 29.76) believed in fortune telling more strongly, *MD* = 12.35, *t* (438.49) = 4.62, *p* < 0.01.

The correlations between beliefs in fortune telling and risk taking across domains are as follows: *r* = .42, *p* < .001, in the ethical domain; *r* = .44, *p* < .001, in the financial domain; *r* = .34, *p* < .001, in the health and safety domain; *r* = .33, *p* < .001, in the recreational domain; *r* = —.13, *p* < .01, in the social domain and *r* = .28, *p* < .001, as measured by the financial risk tolerance test. In sum, belief in fortune telling was positively correlated with risk-taking, except for in the recreational domain.

The descriptive analysis for people’s reported risk taking across different domains are displayed in Table 2. As in Study 1, we conducted an ANCOVA using beliefs in fortune telling as a covariate to test whether participants’ risk taking across different domains would be affected by the fortune telling outcomes that they received; also, as in Study 1, we examined the role of gender.

**Table 2:** Descriptive analysis for risk taking in different domains in Study 2.

Conditions	Gender	*N*	Ethical		Financial		Health & safety	
			*M*	*SD*	*M*	*SD*	*M*	*SD*
Negative	Female	60	24.27	12.88	27.26	12.68	30.24	12.77
	Male	88	35.17	14.56	37.24	13.56	38.87	14.30
	Total	148	30.75	14.86	33.19	14.06	35.37	14.30
Neutral	Female	78	22.87	12.50	25.23	13.24	28.34	12.32
	Male	68	34.51	13.35	35.92	11.60	36.45	13.15
	Total	146	28.28	14.12	30.21	13.56	32.12	13.31
Positive	Female	73	22.11	11.41	25.58	11.88	27.07	11.53
	Male	74	33.29	14.68	36.56	12.41	37.48	10.94
	Total	147	27.74	14.26	31.11	13.30	32.31	12.36
Conditions	Gender	*N*	Recreational		Social		Financial__RT_	
			*M*	*SD*	*M*	*SD*	*M*	*SD*
Negative	Female	60	31.13	15.44	47.54	8.94	15.20	3.75
	Male	88	38.86	14.11	47.39	9.93	16.84	3.26
	Total	148	35.73	15.10	47.45	9.51	16.18	3.55
Neutral	Female	78	27.97	14.44	48.59	8.52	14.50	3.87
	Male	68	39.08	15.06	49.30	8.57	17.41	3.49
	Total	146	33.14	15.70	48.92	8.52	15.86	3.96
Positive	Female	73	29.39	14.87	49.13	9.74	14.27	2.99
	Male	74	39.23	13.11	47.69	8.92	18.12	3.98
	Total	147	34.34	14.81	48.40	9.34	16.21	4.01

Note. Financial__RT_ = financial risk tolerance.

#### Financial risk tolerance

**Fig 2:**
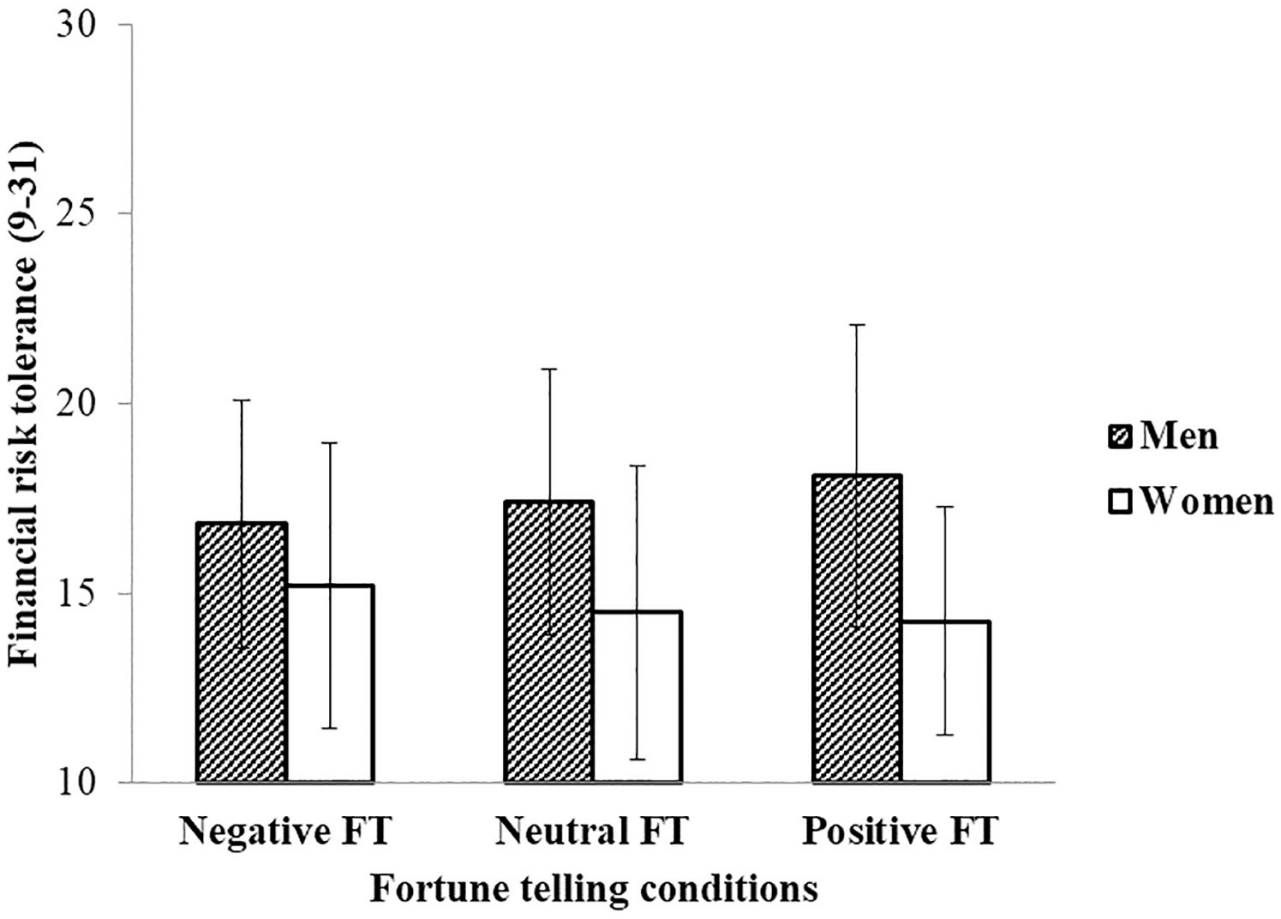
Mean financial risk tolerance among fortune-telling conditions for men and women. Error bars represent standard deviations in Study 2.

Again, beliefs in fortune telling had a significant effect on participants’ financial risk tolerance level, *F* (1, 434) = 18.96, *p* < .001; η_*p*_^2^ = .042. But the main effect of fortune telling outcomes on participants’ financial risk tolerance was not significant, *F* (2, 434) = 0.16, *p* = .850; η_*p*_^2^ = .001. Again, a strong gender difference in financial risk tolerance appeared in this study, *F* (1, 434) = 51.21, *p* < .001; η_*p*_^2^ = .106. Compared to women (*M* = 14.62, *SD* = 3.56), men (*M* = 17.42, *SD* = 3.60) showed higher level of financial risk taking. Again, we found an interaction effect of fortune telling conditions and gender on people’s financial risk tolerance, *F* (2, 434) = 3.41, *p* = .034; η_*p*_^2^ = .015. The interaction pattern of gender and fortune telling conditions on financial risk tolerance is displayed in Fig 2. Consistent with Study 1, there was a trend that the positive prediction increased financial risk tolerance for men. But different from Study 1, the trend for women was that the negative prediction increased financial risk tolerance in Study 2. However, for both men and women, these differences were only marginally significant between positive and negative conditions (for men: *b* = 0.99, *SE* = 0.56, *t* = 1.78, *p* = .075, 95% confidence interval (CI) = [−1.10, 2.09] (See S3 Table in S1 File); for women: *b* = −1.14, *SE* = 0.61, *t* = −1.87, *p* = .063, 95% confidence interval (CI) = [−2.34, 0.06] (See S4 Table in S1 File).

#### Risk taking across domains

As can be seen in Table 3, people’s beliefs in fortune telling was an important predictor for people’s risk-taking behaviors across all the domains. Except for the social domain, the stronger people believed in fortune telling, the more likely they took risks. In addition, gender was an important factor determining people’s risk-taking behaviors. Compared to women, men were more risk-taking across all the domains except in the social domain. Moreover, we found that independent of gender, the fortune telling manipulation had a significant effect on people’s risk taking in the ethical (*F* (2, 434) = 3.36, *p* = .036; η_*p*_^2^ = .015), and health and safety domains (*F* (2, 434) = 3.69, *p* = .026; η_*p*_^2^ = .017), but not in the other domains. Unexpectedly, as can be seen in Table 3, the more negative fortune telling outcome, the higher risk taking in the ethical domain, but only the difference between negative and positive conditions reached significance (*MD* = 3.78, *SE* = 1.45, *t* = 2.59, *p* = .010). Similarly, a negative fortune telling increased people’s risk taking in the health and safety domain versus a neutral condition (*MD* = 3.03, *SE* = 1.41, *t* = 2.11, *p* = .033) and a positive condition (*MD* = 3.61, *SE* = 1.41, *t* = 2.53, *p* = .012).

**Table 3:** ANCOVA for influence of fortune telling manipulation on risk taking in different domains in Study 2.

Variables	Ethical	Financial	Health & safety	Recreational	Social	Financial__RT (Risk tolerance)_
	*F*	*F*	*F*	*F*	*F*	*F*
Beliefs in fortune telling	73.96***	83.26***	45.11***	40.37***	7.64**	18.96***
Gender	53.34***	51.51***	37.66***	30.98***	0.09	51.21***
Fortune telling conditions	3.36*	2.91	3.69*	1.30	1.45	0.16
Gender × Fortune telling	0.10	0.06	0.21	0.60	0.44	3.41*

* *p* < .05;

** *p* < .01;

*** *p* < .001;

Financial__RT_ = financial risk tolerance.

In brief, the link between superstitious beliefs and risk taking was robust across various of domains, and so was the finding that men were more risk taking than women. However, unexpectedly, the effects of the fortune telling manipulation on people’s risk taking were absent in most of the domains and were even in the opposite direction to the predictions in the ethical domain and health and safety domain. Again, we found the interaction of fortune telling with gender on the same financial risk tolerance measure as Study 1, but not on the self-reported financial risk-taking subscale.

#### General self-efficacy

We explored whether the fortune telling manipulation would affect people’s general self-efficacy and hence their risk-taking behaviors. However, beliefs in fortune telling was not a significant predictor of general self-efficacy, *F* (1, 434) = 1.04, *p* = .308; η_*p*_^2^ = .002. The ANCOVA analysis revealed no statistical evidence for the effect of the fortune telling manipulation either, *F* (2, 434) = 1.45, *p* = .236; η_*p*_^2^ = .007. No gender difference in general self-efficacy appeared, *F* (1, 434) = 0.37, *p* = .546; η_*p*_^2^ = .001. No significant interaction effect of manipulation and gender emerged, *F* (2, 434) = 0.04, *p* = .961; η_*p*_^2^ = 0. Furthermore, results revealed that general self-efficacy predicted decreased risk taking in the ethical (*r* = − .22, *p* < .001) and health & safety domains (*r* = − .14, *p* = .002), but increased risk taking in the social domain (*r* = .31, *p* < .001). No significant correlations with general self-efficacy were found in the other risk-taking domains. These findings suggest that the general self-efficacy cannot explain the results presented here.

### Discussion

In Study 2, we used more generally formulated fortune telling predictions and added a self-reported risk-taking scale with multiple life domains to the financial risk tolerance test. The moderation effect of gender appeared again in the financial domain as measured by the financial risk tolerance test. The effect of a positive fortune-telling outcome showed the same pattern for men as in Study 1, although the effect was only marginal. Again, we found men had stronger financial risk tolerance after a positive fortune telling. Importantly, we consistently found that men were more risk taking than women in all life domains except the social domain, and stronger beliefs in fortune telling were related to a stronger willingness to take risks in all life domains except the social domain. The fortune telling manipulation did not influence self-reported risk-taking in different life domains, however. We also found some unexpected findings when risk taking was measured with the self-reported scales. As self-assessment often provides inaccurate self-predictions [38], we wondered whether fortune telling would affect people’s concrete risk-taking behaviors. We examined this in Study 3.

## Study 3: Fortune-telling and gambling behaviors in the laboratory

Study 3 was designed to extend the two online studies in several aspects. First, we examined real risk-taking behavior. To do so, we introduced a gambling game to investigate the influence of fortune telling on people’s real financial risk-taking behaviors. Second, we complemented two online studies by conducting Study 3 in the laboratory, a controlled environment where participants are less likely to be distracted or interrupted. Third, as perceived control can be affected by a certain superstition (e.g., ritual enactment; [18]), and stronger perceived control has been found to be related to a higher level of risk taking [39], in Study 3 we explored whether feelings of control over the future were enhanced by positive fortune telling and were related to stronger financial risk-taking.

In Study 3, we used the same fortune-telling manipulation as in Study 1. After the manipulation, however, we asked participants to play two small gambling games: One was a hypothetical gambling game that involved willingness to buy lottery tickets, and the other one was a real gambling game in which participants got the opportunity to bet a bonus of 50 Euro cents for a 10% chance of winning 5 Euros. We hypothesized that receiving a positive fortune-telling outcome (versus neutral or negative) would make people more likely to play the gambling game. Furthermore, based on the results of Study 1 and 2, we expected that this would be especially true for men. We also included six items to measure participants’ feelings of control over the future to test its relationships with fortune telling and financial risk-taking.

### Method

#### Participants and design

195 participants were recruited on campus to participate in our lab study for 2 Euros or course credits. During the study participants also received a bonus of 50 Euro cents, which they could either keep, or bet with a 10% chance of winning 5 Euros. One male participant was excluded for providing an incomplete response, while one female participant was excluded because she indicated she did not answer all the questions seriously at the end of the survey, leaving a total of 193 valid cases (67.9% female; *M*_age_ = 21.80, *SD* = 4.25; 81.9% Dutch native speakers). Participants were provided with an online link to the experiment hosted on Qualtrics. Participants were randomly assigned to one out of three possible fortune-telling outcomes (positive, neutral, negative).

#### Procedure and measures

The research proposal entitled "The influence of fortune telling on financial risk taking" was approved by the Scientific and Ethical Review Board (VCWE) of the Faculty of Behavior & Movement Sciences, VU University Amsterdam, on 22nd April 2016. Participants filled out informed written consent before taking part in the study and all the data were analyzed anonymously. The procedure was almost the same as in Study 1 except for two differences. First, after rating the fortune telling app, we also asked participants to rate their feelings of control over their future. There were six items in total (α = 0.83). Two example items are “To what extent do you have the feeling that you have some control over your future luck?” and “How much influence do you feel like you have over your future luck?”. These items were measured with 100-point sliders, with which “0” means “Very little control”, while “100” means “A great deal of control”. Second, after ‘Study two’, which contained the same materials from Study 1 and Study 2 for a recognition task as a filler, we told participants to start ‘Study three’, in which we asked participants to play two gambling games. One was a hypothetical gambling game while the other was a real gambling game. In the hypothetical game, we told participants to imagine that from their participant fee, they could buy lottery tickets (25 Euro cents or 2 course credits for each with possibility of winning 10 Euros) with a maximum number of 8. Participants then indicated how many lottery tickets they would buy. (The range of the number of the lottery tickets participants could buy was from 0 to 8).

Game two, then, was a real gambling game. We told participants that every participant would receive an additional 50 Euro cents on top of their fee or credits. We offered them two options: (1) Keep the additional 50 Euro cents. In this case, participants would leave the experiment with 2 Euros or course credits, plus an additional 50 Euro cents; Or (2) play a real small gambling game with the additional 50 Euro cents as a wager. In the latter case, besides the 2 Euros or course credits, participants would have 10% chance of winning an additional 5 Euros and 90% chance of winning nothing. We first asked participants to rate the extent to which they favored each option: Either play or not play using a 100-point scale. Then we asked them to make the final decision whether to play or not. Participants who chose to play the gambling game would either lose the additional 50 Euro cents or win an additional 5 Euros, which was determined by chance (i.e., the 5 Euros were provided in 10% of the cases).

At the end, we rated participants’ beliefs in fortune telling in the same way as it was in Study 1. The correlation between the two items was high, *r* = .61 (*p* < .001), and we averaged participants’ responses into an index of beliefs in fortune telling. All the participants were debriefed at the end of the research.

### Results

#### Manipulation checks

Again, most of the participants (around 80%) were able to accurately recall which fortune telling outcome they had received: 85.9% in the negative condition, 79.7% in the neutral condition, and 86.2% in the positive condition.

#### Beliefs in fortune telling

We conducted a one sample t-test to test the difference between participants’ reported beliefs in fortune telling (*M* = 22.11, *SD* = 21.54) with the scale mid-point of 50. Result revealed that participants’ reported beliefs in fortune telling was significantly lower than the neutral midpoint of the scale, *MD* = − 27.89, *t* (192) = − 17.99, *p* < .001, indicating that in general, participants did not believe in fortune telling. However, in this study we did not find a significant gender difference in beliefs in fortune telling. Women (*M* = 23.26, *SD* = 20.40) and men (*M* = 19.68, *SD* = 23.76) did not differ in their fortune telling beliefs, *MD* = 3.59, *t* (191) = 1.08, *p* = .28.

#### Willingness to buy hypothetical lottery tickets

We aimed to test whether participants’ willingness to buy hypothetical lottery tickets would be affected by the fortune telling predictions they received. As in previous studies, we included beliefs in fortune telling as a covariate into the model. However, beliefs in fortune telling was not a significant predictor for people’s willingness to buy the lottery tickets, *F* (1, 186) = 0.60, *p* = .441; η_*p*_^2^ = .003. It revealed no difference in the extent to which participants were willing to buy lottery tickets between the fortune telling conditions either, *F* (2, 186) = 0.21, *p* = .813; η_*p*_^2^ = .002. We only found a significant gender difference in participants’ willingness to buy lottery tickets, *F* (1, 186) = 7.01, *p* = .009; η_*p*_^2^ = .036. Male participants were willing to buy more lottery tickets (*M* = 4.06, *SD* = 3.13) than female participants (*M* = 2.98, *SD* = 2.41). The interaction of fortune telling conditions with gender was not significant, *F* (2, 186) = 0.10, *p* = .909; η_*p*_^2^ = .001.

#### Real gambling behavior

The correlation between real gambling decision-making and the number of hypothetical lottery tickets participants were willingness to buy was moderate (*r* = .31, *p* < .001). It suggests that it is useful to run separate analyses on the hypothetical gamble and the real gamble for actual money. In a generalized linear model, choosing “Binomial” for probability distribution, “Logit” for link function, and treating “1” (betting) as the response, while treating “0” (not betting) as the reference category, we checked the effects of beliefs in fortune telling, gender, and the interaction effects of gender and fortune telling conditions on participants’ decision making for the gambling game. The model was satisfactory, which was marginally significant against the intercept-only model, χ^2^ (6, *N* = 193) = 11.37, *p* = .078.

Surprisingly, the effect of beliefs in fortune telling and the effect of gender on participants’ decision-making for a gambling game were not significant, χ^2^ (1, *N* = 193) = 1.63, *p* = .202 and χ^2^ (1, *N* = 193) = 1.33, *p* = .249 respectively. Although it showed men (95.5%) were more likely to gamble as compared to women (76.7%) after a positive fortune telling outcome, the interaction effect of gender and fortune telling did not reach significance in this study, χ^2^ (2, *N* = 193) = 3.27, *p* = .20.

**Fig 3:**
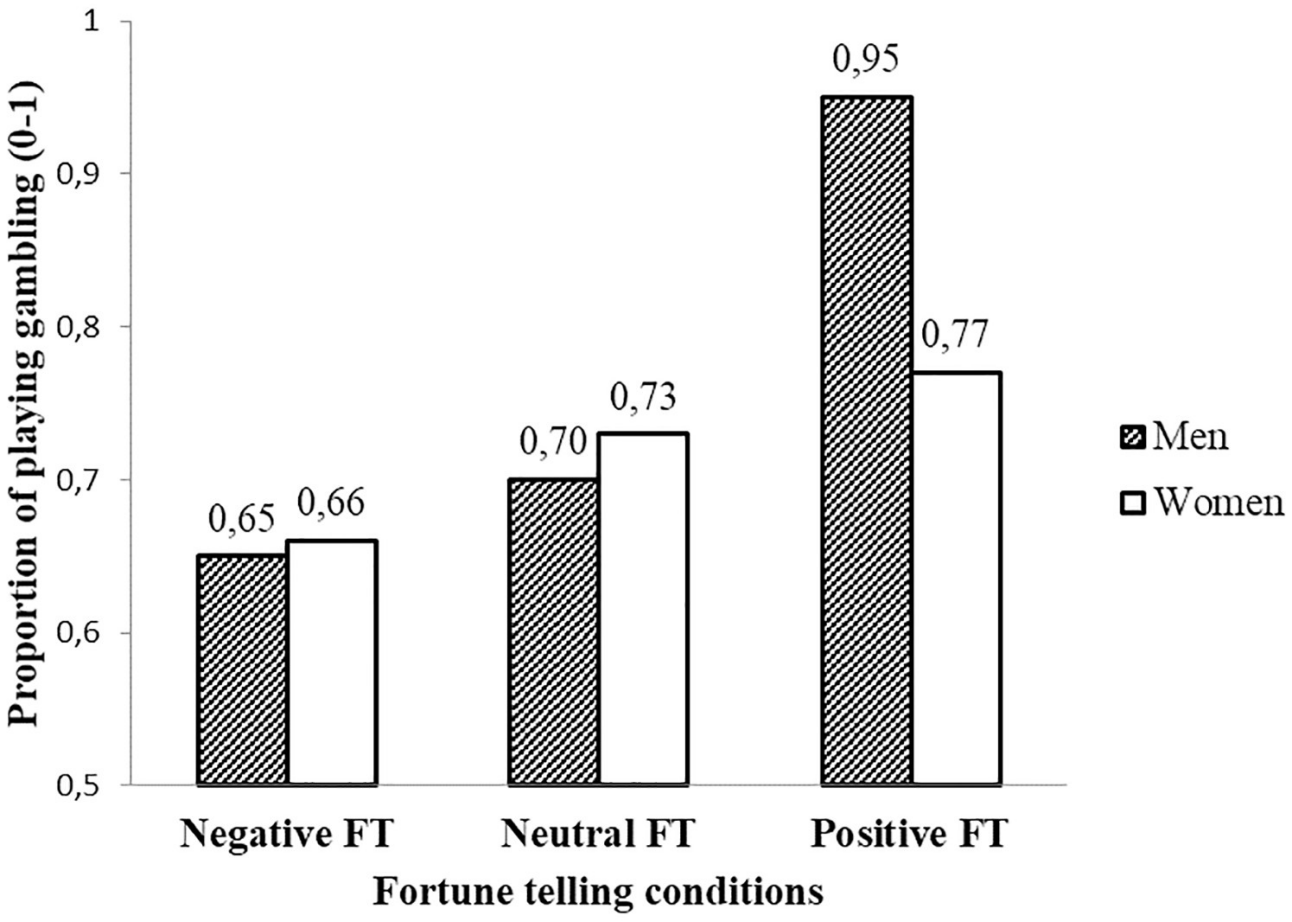
Proportion of playing a gambling game among fortune telling conditions for men and women in Study 3.

However, as in Study 1, we found a significant main effect of the fortune telling manipulation, χ^2^ (2, *N* = 193) = 6.66, *p* = .036. The percentage of choosing to gamble in each fortune telling condition (for men, women, and total) is listed in Table 4 and Fig 3. According to multiple comparisons of estimated marginal means with Bonferroni adjustment, the more positive fortune telling outcome participants received, the more likely participants decided to gamble, with 90% of choosing to gamble in the positive condition, as compared to 71% in the neutral condition, *MD* = 0.19, *SE* = 0.08, *p* = .046, 95% confidence interval (CI) = [0, 0.38]; and 64% in the negative condition, *MD* = 0.26, *SE* = 0.08, *p* < .01, 95% confidence interval (CI) = [0.06, 0.46]. The difference between negative and neutral conditions was not significant. In sum, positive fortune telling leads to more gambling, but only in the case of a real gamble, not a hypothetical one. This effect was not moderated by gender in the present study; put differently, as in the previous studies also in Study 3 positive fortune telling increased financial risk-taking among men; but, in this study the effect also emerged among women.

**Table 4:** Descriptive statistics for decision-making for a real gambling game among different fortune telling conditions in Study 3.

		Decision-making (Count (%))		Total
		Not to gamble	To gamble	
Negative condition	Male	7(35%)	13(65%)	20
	Female	15(34.1%)	29(65.9%)	44
	Subtotal	22	42	64
	% within conditions	34.4%	65.6%	100%
Neutral condition	Male	6(30%)	14(70%)	20
	Female	12(27.3%)	32(72.7%)	44
	Subtotal	18	46	64
	% within conditions	28.1%	71.9%	100%
Positive condition	Male	1(4.5%)	21(95.5%)	22
	Female	10(23.3%)	33(76.7%)	43
	Subtotal	11	54	65
	% within Conditions	16.9%	83.1%	100%
Total	Count	51	142	193
	% within conditions	26.4%	73.6%	100%

#### Feelings of control over future

We explored whether the fortune telling manipulation affected people’s feelings of control over the future and hence affected their risk-taking behaviors. However, there was no effect of fortune telling on people’s feelings of control over future, *F* (2, 186) = 1.74, *p* = .179. The interaction with gender was not significant either, *F* (2, 186) = 1.73, *p* = .181. Meanwhile, there was almost no correlation between feelings of control over future and risk-taking behaviors in this study. These findings indicate that the effects of fortune telling on risk taking emerge independent of participants’ feelings of control over future.

### Discussion

Again, Study 3 suggested that participants mostly did not believe in fortune telling. Unexpectedly, the relationship between beliefs in fortune telling and financial risk taking did not reach statistically significance. No significant effect of fortune telling conditions was found on participants’ willingness to buy hypothetical lottery tickets. One possible reason for this finding is that participants were not sensitive to the hypothetical payoffs. The importance of using real cash payoffs was also discussed by Holt and Laury [40]. At the same time, Study 3 did provide further evidence, for the enhancing effect of a positive fortune telling on people’s financial risk-taking behavior as reflected in actual gambling behavior in a lab setting. A randomly assigned positive fortune-telling outcome made participants more likely to play an actual gambling game rather than keep the money for themselves. This effect was independent of gender, while the gender difference in gambling did not show up either. Finally, we explored whether feelings of control played a role in the effects of fortune telling. However, no evidence supported this speculation.

## Meta-analysis: Gender as a moderator for the effect of positive fortune telling

Across three experimental studies, we consistently found that positive fortune telling (either versus neutral fortune telling or negative fortune telling) enhanced men’s financial risk taking. However, the effects for women were less consistent. The Study 2 findings suggest that these interactive effects of gender are specific for financial risk-taking and does not generalize to other domains through self-report scales. Since the moderation effect of gender was not significant in Study 3 and only marginally significant in Study 1, and the simple contrasts of positive fortune telling condition versus neutral condition were not statistically significant in Study 1 and Study 2, we conducted an internal meta-analysis recommended by some researchers [41] to examine the robustness of the moderation effect of gender and the simple contrast effects of positive fortune telling against a neutral condition for men and women respectively.

We calculated the effect sizes and sampling variances for the contrast of a positive fortune telling condition against a neutral condition for men and women respectively in three studies using the *R* package ‘Effect Size Computation for Meta Analysis’ [42]. In order to use the same measure of effect size across three studies, we followed a conventional way [43] to have transformed log (Odds Ratio) and sampling variances in Study 3 into standardized mean difference and new sampling variances, and we used the latter for Study 1 and Study 2. We first tested the moderation effect of gender using the R package ‘metafor’ [44]. We found that the moderation effect of gender was significant, *QM* (*df* = 1) = 4.47, *p* = .035. Then we estimated the average effect size for men and women. The meta-analysis revealed that the effect size (SMD, standardized mean difference) for men was 0.33, 95% confidence interval (CI) = [0.08, 0.58], which indicates a small to medium effect, whereas there was no significant effect for women, the estimate of which was– 0.05, 95% confidence interval (CI) = [– 0.30, 0.20].

We also conducted an internal meta-analysis to examine gender as a moderator for the effect of negative fortune telling (versus the neutral control condition). We found that the moderation effect of gender was not significant, *QM* (*df* = 1) = 0.003, *p* = .958. The effect size (SMD, standardized mean difference) for women was almost equal to zero, the estimate of which was –0.07, 95% confidence interval (CI) = [–0.31, 0.18], whereas it was also almost equal to zero (–0.06, 95% confidence interval (CI) = [–0.31, 0.20]) for men as well. To conclude, no solid evidence was found for an effect of a negative fortune telling outcome (versus the neutral control condition) neither on men’s nor on women’s financial risky decision-making.

## General discussion

Three experiments and a meta-analysis uncovered three main findings. First, findings revealed a positive association between superstitious beliefs in fortune telling and financial risk taking. People who reported higher superstitious beliefs in fortune telling also reported an increased tolerance for financial risks (found in Studies 1 and 2). Second, in general participants indicate that they do not believe in fortune telling (across three studies). Third, despite the fact that they did report not to believe in fortune telling, participants (especially men) nevertheless were affected by it: Positive fortune telling enhances men’s financial risk taking (across three studies), whereas no such effect of positive fortune telling emerged among women (except in Study 3). This risk-taking effect for men in the financial domain as consequence of a positive fortune telling was further supported by a meta-analysis of the three experiments.

### The paradox–not believing it but acting upon it

Our research findings are consistent with [13]’s claim that people often are susceptible to superstition even when they claim to not believe in it. There is a paradox about believing in superstition in modern times—that is, people act upon superstition while they claim to not believe in it. Since the rise of scientific empiricism, superstition has been negatively valued in society. For instance, it was believed that superstition was caused by the workings of a lower form of human intelligence [45]. New insights produced a shift in common understandings of superstition, however. As various scholars have suggested, it may be part of human nature to construct causal relationships among events, regardless of whether the causal links are real or not (e.g., [12, 16]). Specifically, it is common for people to be superstitious by believing that luck (good or bad) is controllable or predictable, even if they do not admit to it. Across three studies, we found in general, people claimed that they do NOT believe in fortune telling, but nevertheless, particularly men’s financial decisions were affected by the (positive) fortune telling outcomes. Unless people know that they do not want to admit to it, these findings provide novel but indirect evidence that superstition may exert influence on people at a subconscious level.

### Gender difference in superstition

There is no scientific consensus yet regarding the question if men or women are more superstitious. Some researchers proposed that women are more superstitious [46]. However, some research evidence is consistent with the notion that *men* are more superstitious. For instance, a field experiment on lucky numbers suggested that being assigned to lucky numbers does not influence women but increases overconfidence among men [34]. In two of our three studies (Study 1 and 2) we found that men have stronger beliefs in fortune telling. In addition, according to the meta-analytic overview of three studies, men were significantly affected by positive fortune telling whereas women were not. The present research therefore supports the idea that men are more susceptible to superstition than women, at least in financial decision-making situations.

### Superstition and risk taking

In two of our three studies (Study 1 and 2) we found significant positive associations between superstition and risk taking. These findings are consistent with the hypothesis that superstition works as a coping-mechanism in a risky decision-making situation. From this point of view, the riskier the situation, the stronger people’s need for superstition. Some groups, including sportsperson, gamblers, sailors, soldiers, miners, financial investors and college students, who have to deal with high-risk situations are also considered as traditionally superstitious groups [1]. Additionally, superstitious beliefs were found to be correlated with gambling intensity among EGM (electronic gaming machine) gamblers [47]. A recent study [48] also suggests that the presence of religious images tends to increase individuals’ subjective probability of winning the lottery, and that subjects therefore believe in a god who intervenes actively in the world in response to their requests.

This point of view may also help people understand why men are more affected by a positive fortune telling. According to a meta-analysis [33], men in general are more risk taking than women. Men consistently take greater risks than women in the financial domain [49, 50]. From an evolutionary perspective, risk-taking behaviors may serve multiple important functions for men, such as acquiring social status and resources, attracting high-quality mates, and establishing and affirming manhood after gender threats (for a review, see [51]). However, risks naturally entail potential losses, and thereby pose potential threats to risk-takers. Men are not totally blind to risks. Superstition suggesting that good luck is ahead may increase men’s expectations of “beating the odds”, decrease anxiety among men, provide a justification for a risky choice, and consequently increase their risk-taking behaviors. Put differently, men are more likely than women to take risks in the financial domain, and positive superstitious beliefs encourage such risk-taking further.

### Strengths, limitations, and future directions

At least two strengths are noteworthy. First, as noted earlier, research on the effects of superstition on financial risk taking constitutes largely uncharted territory [21], and thus the present research is filling a gap in the literature on superstition and decision-making. We regard this as an important gap also because it adds to the literature that seemingly irrational factors do affect decision-making, even in a high-status domain (financial decision-making) that many people might associate with logical analysis and precision. Second, most empirical research on this topic was conducted with Asian participants (e.g., [25]), who are from cultures where superstition and rituals are slightly more common. The present research was conducted with Western samples, which may add to the generalizability of the findings on superstition.

We also want to discuss several limitations of the current research. One limitation is that because the empirical study of superstition is quite novel and theories of superstition are still rather preliminary, the present research is mostly driven by assumptions about the underlying processes that cause these effects. We believe the findings reported may therefore contribute to theorizing illuminating why men are subject to superstition when they take financial risks. Another limitation pertains to the scope of the superstition construct that we investigated. Specifically, we only investigated one form of superstition, which is fortune telling. It cannot be concluded with confidence whether the findings will replicate for other forms of superstition. Moreover, the saliency of the payoffs in the experimental study conducted in the lab was relatively low, as compared to the payoffs in a typical economic experiment, and the overall financial incentives for all the participation were rather low as well. According to [52, 53], risk aversion increases sharply when payoffs are scaled up. These low payoffs, particularly the small stakes of mild risks for the gambling decision-making in Study 3 with a fixed payment of 2 euros for both options, could have affected the ecological validity of the studies. But however, throughout history, kings and generals customarily called on astrologists or fortune-tellers to obtain advice prior to making any important decisions (e.g., launching a military campaign) [54], it is a question whether the risk-enhancement effect of a positive fortune telling outcome would be present or absent for a real-life decision making involving big stakes. This suggests worthwhile empirical challenges for further research. Besides, the possibility that it is the “positivity” of the message that actually drives men to take more risks under a positive fortune telling condition cannot be ruled out with the present research. This could be investigated in future research on the effects of fortune telling on people’s risk seeking. Finally, there may be validity issues on the measurement instruments used for the present research. While some of our measures were commonly used by other researchers (e.g., the financial risk tolerance test), other measurement instruments have not been validated in prior research (e.g., the items for beliefs in fortune telling). The items of these scales did directly ask for the constructs of interest, however, substantially minimizing potential threats to the construct validity for these measurement instruments.

## Conclusions

In spite of the popularity of superstition in people’s daily life, theories and empirical research on people’s susceptibility to superstition are still rather limited. The present findings indicate that positive fortune telling is an important factor for people’s financial risk taking. This seems particularly true for men, even when they claim not to believe in superstition. This paradox is intriguing in itself, and potentially important to understand people’s behaviors as financial decision makers–at home, in organizations, or at financial markets. As such, the findings add credence to the general idea that relatively subtle processes, which decision makers may not be able or willing to recognize, can exert quite pronounced influences in taking financial risks.

## Supporting information

S1 File (PDF)
