# Supplementary material for: Positive fortune telling enhances men’s financial risk taking
Source: PLoS One. 2022 Sep 7;17(9):e0273233. doi: 10.1371/journal.pone.0273233 (PMC9451074; doi:10.1371/journal.pone.0273233)
Supplement: S1 File — (PDF) [file pone.0273233.s001.pdf]

Table of Contents

Simple Slopes Tests by Regression Analyses for Study 1 and Study 2 2
Table 1 2
Simple Slopes Tests by Regression Analysis for Study 1 (Male was set as the reference category) 2
Table 2 2
Simple Slopes Tests by Regression Analysis for Study 1 (Female was set as the reference category) 2
Table 3 3
Simple Slopes Tests by Regression Analysis for Study 2 (Male was set as the reference category) 3
Table 4 3
Simple Slopes Tests by Regression Analysis for Study 2 (Female was set as the reference category) 3
Methodology Information for Three Positive Fortune Telling Studies 4
Study 1 4
General instructions 4
Fortune telling manipulation 4
Perception tasks 6
Financial risk tolerance assessment 7
Measure for beliefs in superstition 10
Study 2 10
General instructions 10
Fortune telling manipulation 10
Perception tasks 11
Measure of risk taking across different domains 11
Financial risk tolerance assessment (the same as in Study 1.) 12
A general self-efficacy scale 12
Measure for beliefs in superstition (the same as in Study 1.) 12
Study 3 12
General instructions 12
Fortune telling manipulation 12
Perception test 13
Fortune telling manipulation 13
Measure for feelings of control over future (Self-made) 13
Gambling games 13
Measure for beliefs in superstition (the same as in Study 1 and Study 2.) 14
References 14

Simple Slopes Tests by Regression Analyses for Study 1 and Study 2

Table 1

Simple Slopes Tests by Regression Analysis for Study 1 (Male was set as the reference category)

Model	Unstandardized Coefficients	SE	t	p value
Constant	15.32	0.57	26.84	.000
Beliefs in fortune telling	0.04	0.01	4.60	.000
Gender (Male = 0)	−0.40	0.78	−0.52	.607
Fortune telling conditions				
D1 (Positive fortune telling)	1.97	0.69	2.86	.005
D2 (Negative fortune telling)	0.21	0.73	0.29	.770
Fortune telling conditions by Gender				
D1 by Gender	−2.43	1.06	−2.28	.023
D2 by Gender	−2.07	1.07	−1.93	.054

Note. One dummy variable was computed for "Gender", and Male was set as the reference category. Two dummy variables (D1 = Positive fortune telling and D2 = Negative fortune telling) were computed for the "Fortune telling conditions", and Neutral fortune telling condition was set as the reference category.

Table 2

Simple Slopes Tests by Regression Analysis for Study 1 (Female was set as the reference category)

Model	Unstandardized Coefficients	SE	t	p value
Constant	14.92	0.64	23.27	0.000
Beliefs in fortune telling	0.04	0.01	4.60	0.000
Gender (Female = 0)	0.40	0.78	0.52	0.607
Fortune telling conditions				
D1 (Positive fortune telling)	−0.45	0.81	−0.56	0.576
D2 (Negative fortune telling)	−1.86	0.79	−2.35	0.020
Fortune telling conditions by Gender				
D1 by Gender	2.43	1.06	2.28	0.023
D2 by Gender	2.07	1.07	1.93	0.054

Note. One dummy variable was computed for "Gender", and Female was set as the reference category. Two dummy variables (D1 = Positive fortune telling and D2 = Negative fortune telling) were computed for the "Fortune telling conditions", and Neutral fortune telling condition was set as the reference category.

Table 3

Simple Slopes Tests by Regression Analysis for Study 2 (Male was set as the reference category)

**Model**	***Unstandardized Coefficients***	***SE***	***t***	***p value***
Constant	16.83	0.37	45.19	.000
Beliefs in fortune telling	0.03	0.01	4.35	.000
Gender (Male = 0)	−1.32	0.59	−2.23	.026
Fortune telling conditions				
D1 (Positive fortune telling)	0.99	0.56	1.78	.075
D2 (Neutral fortune telling)	0.42	0.57	0.75	.456
Fortune telling conditions by Gender				
D1 by Gender	−2.13	0.82	−2.59	.010
D2 by Gender	−1.31	0.82	−1.59	.114

Note. One dummy variable was computed for “Gender”, and Male was set as the reference category. Two dummy variables (D1 = Positive fortune telling and D2 = Neutral fortune telling) were computed for the “Fortune telling conditions”, and Negative fortune telling condition was set as the reference category.

Table 4

Simple Slopes Tests by Regression Analysis for Study 2 (Female was set as the reference category)

**Model**	***Unstandardized Coefficients***	***SE***	***t***	***p value***
Constant	15.51	0.46	33.98	.000
Beliefs in fortune telling	0.03	0.01	4.35	.000
Gender (Female = 0)	1.32	0.59	2.23	.026
Fortune telling conditions				
D1 (Positive fortune telling)	−1.14	0.61	−1.87	.063
D2 (Neutral fortune telling)	−0.88	0.60	−1.47	.143
Fortune telling conditions by Gender				
D1 by Gender	2.13	0.82	2.59	.010
D2 by Gender	1.31	0.82	1.59	.114

Note. One dummy variable was computed for “Gender”, and Female was set as the reference category. Two dummy variables (D1 = Positive fortune telling and D2 = Neutral fortune telling) were computed for the “Fortune telling conditions”, and Negative fortune telling condition was set as the reference category.

Methodology Information for Three Positive Fortune Telling Studies

Study 1

General instructions

“Welcome!

These are three different small studies that call for your participation. The first study will test people's satisfaction with an easy app developed for predicting people's future fortune based on demographic information, such as birth date. The second study will test people's recognition of six common materials, including leather, glass, metal, paper, plastic and fabric. The third study will assess how people generally make their financial decisions. Together, these three studies will take about 15 minutes to complete.”

Fortune telling manipulation

Instructions:

“In this study you are asked to provide specific information about your birth and your favorite color. Based on this information, our software can match it with those people who are mostly similar to you in our data set (having recorded 20,000,000 people's information) and tell you how fortunate your future might be. Please provide information about your birth and your favorite color as accurately as possible when you answer the following questions. All your information you provide will be kept confidential and anonymous.

In which year were you born?

Year

In which month were you born?

Month

Day

At what time were you born? (Estimate it if you are not sure about it.)

Hour

Minute

In which continent were you born?

Continent

Which color listed below is your favorite color?

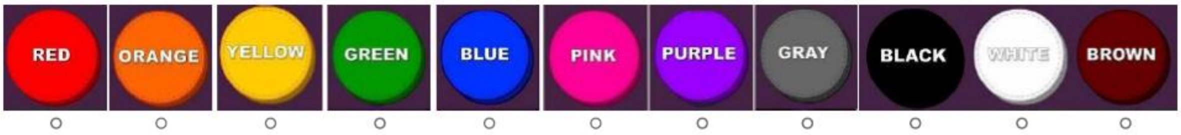

Your information is successfully recorded by the app system, please click 'Submit' to submit it to the system, then you can get your report. As the internet speed varies from time to time, it may take a while to get your report ready. Please wait patiently. Once you have read your report, please click 'Next' to move to the next page, where you are asked to rate your personal experience of using this app. Many thanks for your cooperation in advance!”

Prediction given in the neutral fortune telling condition*:*

There is only a prediction about personality traits:

"According to the estimation from our app, you are a reliable, charismatic and interesting person. Sometimes, you can be serious, thoughtful and even indecisive. These qualities will continue to influence your life in many ways, both in the near and distant future."

Prediction given in the positive fortune telling condition:
Besides the prediction about personality traits, which is the same as in the neutral condition, there is a specific positive prediction:
"The app predicts that you will have a lot of luck in the financial domain. Chances are big that you will always have enough money for a high-quality life."

Prediction given in the negative fortune telling condition:
Besides the prediction about personality traits, which is the same as in the neutral condition, there is a specific positive prediction:
"The app predicts that you will have a lot of luck in the financial domain. Chances are big that you will always have enough money for a high-quality life."

App rating questions
Instructions:
"You are back to the questionnaire. Please rate your experience of using the app being tested with a 7-point likert scale from 'extremely dissatisfied' to 'extremely satisfied'."
Items:
To what extent are you satisfied with the ease of use of this app?
To what extent are you satisfied with the prediction from this app regarding your personality traits?
To what extent are you satisfied with the prediction from this app regarding your future fortune state? (*This item is not present in the neutral fortune telling condition*.)

Perception tasks

Instructions:

"In this second part of the research, you are asked to categorize the material you see from each image to the right category. There are six categories of materials in total, including leather, glass, metal, paper, plastic and fabric. We will present you 24 images of different materials, each of which can be categorized into one of these six categories. Each image will last only for five seconds. You need to click the right category for each image as soon as possible within these five seconds. The reaction time will be recorded automatically by the computer. Please click 'Start' to start this task."

One example item:

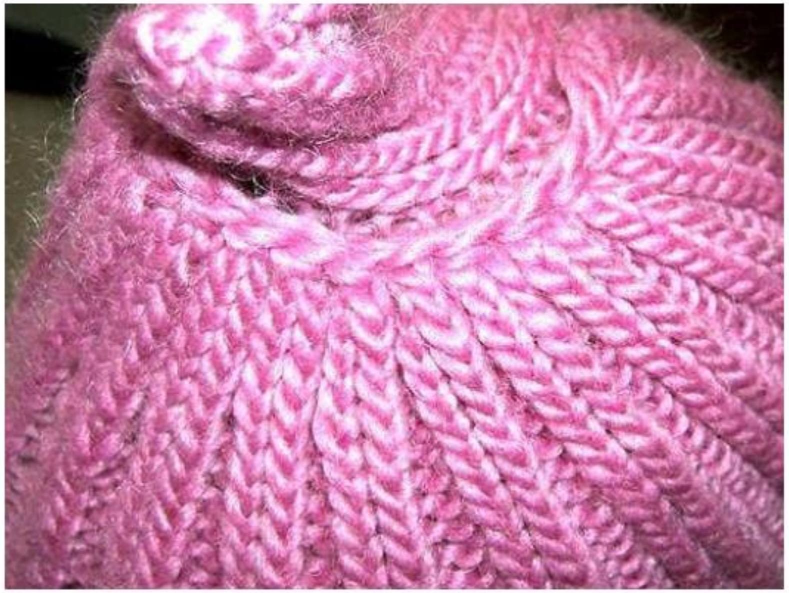

Leather ○ Glass ○ Metal ○ Paper ○ Plastic ○ Fabric ○

Financial risk tolerance assessment

(Note. More details about this financial risk tolerance assessment can be found in the article by Grable and Lytton [1].)

Instructions:

"In this third part of the research, you are asked to fill out a quiz about financial decision making. Choose the response that best describes you – there are no "right" or "wrong" answers. Note: Your results will be recorded anonymously. We are not collecting any identifying information. Please click 'Next' to move to the next page."

Items:

Q1: **Given the best and worst case returns of the four investment choices below, which would you prefer?**

- ○ $200 gain best case; $0 gain/loss worst case (1)
- ○ $800 gain best case; $200 loss worst case (2)
- ○ $2,600 gain best case; $800 loss worst case (3)
- ○ $4,800 gain best case; $2,400 loss worst case (4)

Q2 : If you had to invest $20,000, which of the following investment choices would you find most appealing?

- ○ 60% in low-risk investments 30% in medium-risk investments 10% in high-risk investments (1)
- ○ 30% in low-risk investments 40% in medium-risk investments 30% in high-risk investments (2)
- ○ 10% in low-risk investments 40% in medium-risk investments 50% in high-risk investments (3)

Q3: **If you unexpectedly received $20,000 to invest, what would you do?**

- ○ Deposit it in a bank account, money market account, or an insured CD (1)
- ○ Invest it in safe high-quality bonds or bond mutual funds (2)
- ○ Invest it in stocks or stock mutual funds (3)

Q4: In terms of experience, how comfortable are you investing in stocks or stock mutual funds?

- ◯ Not at all comfortable (1)
- ◯ Somewhat comfortable (2)
- ◯ Very comfortable (3)

Q5: **Some experts are predicting prices of assets such as gold, jewels, collectibles, and real estate (hard assets) to increase in value; bond prices may fall, however, experts tend to agree that government bonds are relatively safe. Most of your investment assets are now in high interest government bonds. What would you do?**

- ◯ Hold the bonds (1)
- ◯ Sell the bonds, put half the proceeds into money market accounts, and the other half into hard assets (2)
- ◯ Sell the bonds and put the total proceeds into hard assets (3)
- ◯ Sell the bonds, put all the money into hard assets, and borrow additional money to buy more (4)

Q6: When you think of the word "risk" which of the following words comes to mind first?

- ◯ Loss (1)
- ◯ Uncertainty (2)
- ◯ Opportunity (3)
- ◯ Thrill (4)

Q7: **You are on a TV game show and can choose one of the following. Which would you take?**

- $1,000 in cash (1)
- A 50% chance at winning $5,000 (2)
- A 25% chance at winning $10,000 (3)
- A 5% chance at winning $100,000 (4)

Q8: **You have just finished saving for a "once-in-a-lifetime" vacation. Three weeks before you plan to leave, you lose your job. You would:**

- Cancel the vacation (1)
- Take a much more modest vacation (2)
- Go as scheduled, reasoning that you need the time to prepare for a job search (3)
- Extend your vacation, because this might be your last chance to go first-class (4)

Q9: **Your trusted friend and neighbor, an experienced geologist, is putting together a group of investors to fund an exploratory gold mining venture. The venture could pay back 50 to 100 times the investment if successful. If the mine is a bust, the entire investment is worthless. Your friend estimates the chance of success is only 20%. If you had the money, how much would you invest?**

- Nothing (1)
- One month's salary (2)
- Three month's salary (3)
- Six month's salary (4)

(***Note***: *Answer choices for each item were given a weight (from 1 to 4) according to the riskiness of the response. Higher weightings indicated a riskier choice, whereas lower weightings indicated a less risky choice. These weight figures were not shown to the participants*.)

Measure for beliefs in superstition

"To what extent do you believe in the prediction given by the fortune telling app? Please rate it with a slider where 0 means 'Not at all' and 100 means 'A great deal'.
The extent I believe in the prediction… (0, 10, 20, 30…100)"

"In general, how credible do you find predictions given by fortune tellers?
Please rate it with a slider where 0 means 'Not at all' and 100 means 'A great deal'.
The credibility I find is... (0, 10, 20, 30…100)".

Study 2

General instructions

"Welcome!
These are three different small studies that call for your participation. The first study will test people's satisfaction with an easy app developed for predicting people's future
fortune based on demographic information, such as birth date. The second study will test people's recognition of six common materials, including leather, glass, metal, paper,
plastic and fabric. The third study will assess how people generally make their decisions in their daily life. Together, these three studies will take about 15 minutes to complete."

Fortune telling manipulation

All the relevant information of this part is the same as in Study 1, except the specific descriptions of the fortune telling outcomes in each fortune telling condition.

Prediction given in the neutral fortune telling condition*:*
As in Study 1, there is only a prediction about personality traits:
"According to the estimation from our app, you are a reliable, charismatic and interesting person. Sometimes, you can be serious, thoughtful and even indecisive."

Prediction given in the positive fortune telling condition:
Besides the prediction about personality traits, which is the same as in the neutral condition, there is a specific positive prediction:
"The app predicts that you will be lucky enough to have a prosperous life in the future. You will always have the opportunity to express your talent and can achieve remarkable success. Chances are big that there will always be enough money for a high-quality life. Meanwhile, you will easily acquire many good friends and have high quality close relationships throughout your life."

Prediction given in the negative fortune telling condition:
Besides the prediction about personality traits, which is the same as in the neutral condition, there is a specific positive prediction:
"The app predicts that you will not be lucky enough to have a prosperous life in the future. You will not always have the opportunity to express your talent and cannot achieve remarkable success. Chances

are small that there will always be enough money for a high-quality life. Meanwhile, you will not easily acquire many good friends and will have no or troublesome close relationships throughout your life."

Perception tasks

This part is the same as in Study 1.

Measure of risk taking across different domains

(Note. More details about this measure can be found in the article by Blais and Weber [2])

Instructions:

"In this third part of the research, you are asked to fill out a quiz about decision making in different life domains. Choose the response that best describes you – there are no "right" or "wrong" answers.
Note: Your results will be recorded anonymously. We are not collecting any identifying information.
Please click 'Next' to move to the next page."

"For each of the following statements, please indicate the likelihood that you would engage in the described activity or behavior if you were to find yourself in that situation. Provide a rating from Extremely Unlikely to Extremely Likely, using the following scale."

Extremely unlikely	Moderately unlikely	Somewhat unlikely	Not sure	Somewhat likely	Moderately likely	Extremely likely

Items:

1. Admitting that your tastes are different from those of a friend.
2. Going camping in the wilderness.
3. Betting a day's income at the horse races.
4. Investing 10% of your annual income in a moderate growth diversified fund.
5. Drinking heavily at a social function.
6. Taking some questionable deductions on your income tax return.
7. Disagreeing with an authority figure on a major issue.
8. Betting a day's income at a high-stake poker game.
9. Having an affair with a married man/woman.
10. Passing off somebody else's work as your own.
11. Going down a ski run that is beyond your ability.
12. Investing 5% of your annual income in a very speculative stock.
13. Going whitewater rafting at high water in the spring.
14. Betting a day's income on the outcome of a sporting event.
15. Engaging in unprotected sex.
16. Revealing a friend's secret to someone else.
17. Driving a car without wearing a seat belt.
18. Investing 10% of your annual income in a new business venture.
19. Taking a skydiving class.

20. Riding a motorcycle without a helmet.
21. Choosing a career that you truly enjoy over a more secure one.
22. Speaking your mind about an unpopular issue in a meeting at work.
23. Sunbathing without sunscreen.
24. Bungee jumping off a tall bridge.
25. Piloting a small plane.
26. Walking home alone at night in an unsafe area of town.
27. Moving to a city far away from your extended family.
28. Starting a new career in your mid-thirties.
29. Leaving your young children alone at home while running an errand.
30. Not returning a wallet you found that contains $200.

Financial risk tolerance assessment (the same as in Study 1.)

A general self-efficacy scale

(Note. More details about this scale can be found in the article by Schwarzer and Jerusalem [3])

Items:

1. I can always manage to solve difficult problems if I try hard enough.
2. If someone opposes me, I can find the means and ways to get what I want.
3. It is easy for me to stick to my aims and accomplish my goals.
4. I am confident that I could deal efficiently with unexpected events.
5. Thanks to my resourcefulness, I know how to handle unforeseen situations.
6. I can solve most problems if I invest the necessary effort.
7. I can remain calm when facing difficulties because I can rely on my coping abilities.
8. When I am confronted with a problem, I can usually find several solutions.
9. If I am in trouble, I can usually think of a solution.
10. I can usually handle whatever comes my way.

Measure for beliefs in superstition (the same as in Study 1.)

Study 3

General instructions

"Welcome!

These are three different small studies that call for your participation. The first study will test people's satisfaction with an easy app developed for predicting people's future fortune based on demographic information, such as birth date. The second study will test people's recognition of six common materials, including leather, glass, metal, paper, plastic and fabric. The third study will focus on people's decision-making for small gambling games. Together, these three studies will take about 15 minutes to complete."

Fortune telling manipulation

All the relevant information of this part is the same as in Study 1.

Perception test

All the relevant information of this part is the same as in Study 1 and Study 2.

Fortune telling manipulation

All the relevant information of this part is the same as in Study 1.

Measure for feelings of control over future (Self-made)

Instructions:

"As part of the second study, here are some questions about your general feelings about your future. Please answer the following questions with sliders, where 0 means very little while 100 means a great deal of."

Items:

1.To what extent do you have the feeling that you have some control over your future luck?

2.How much influence do you feel like you have over your future luck?

3.To what extent do you have the feeling that you are the master of your own life?

4.To what extent do you have the feeling that your wishes are likely to come true?

5.To what extent do you have the feeling that your life is likely to go on in the right direction?

6.To what extent do you have the feeling that your life is under your control?

Gambling games

Instructions:

"In this third part of the research, you are asked to play two small gambling games. Please click 'Next' to move to the next page."

Hypothetical gambling game

"Game one: As you know, you will get 2 euros or 16 course credits for sure for your participation in this research. Suppose as a bonus, you are granted an opportunity to buy our lottery tickets (25 euro cents or 2 course credits for each with possibility of winning 10 euros) with a maximum number of 8. How many lottery tickets would you like to buy?"

0	1	2	3	4	5	6	7	8
○	○	○	○	○	○	○	○	○

Real gambling game

"Game two: This is a real gambling game. In order to reward your participation in this research, the experimenters have decided to give every participant of this research an extra 50 euro cents. With regard to this extra 50 euro cents, we offer you two options: (1) Keep the extra 50 euro cents. In this case, you leave with 2 euros or 16 course credits plus an additional 50 euro cents. Or (2) play our real small gambling game with the extra 50 euro cents. In the latter case, besides the 2 euros or 16 course

credits, you have 10% chance of winning an extra 5 euros and 90% chance of losing the extra 50 euro cents. Would you like to play this gambling game?

"Please indicate the extent to which you would favor option one: Keep the extra 50 euro cents. In this case, you leave with 2 euros or 16 course credits plus an additional 50 euro cents. (100-point scale, 0 means 'No preference at All' and 'Extremely Prefer for Option One')

Please indicate the extent to which you would favor option two: Play the small gambling game with the extra 50 euro cents. In the latter case, besides the 2 euros or 16 course credits, you have 10% chance of winning an extra 5 euros and 90% chance of losing the extra 50 euro cents. (100-point scale, 0 means 'No preference at All' and 'Extremely Prefer for Option Two')

Please make your final decision below. Would you like to play this small gambling game, by which you have 10% chance of winning an extra 5 euros and 90% chance of losing the extra 50 euro cents? Or you would like to just keep this 50 euro cents?

I would like to bet this extra 50 euro cents for 5 euros	I would like to just keep this extra 50 euro cents.
○	○

When you click 'Start', the gambling game will be activated automatically. The outcome of the game will appear immediately. You either win an extra 5 euros, or lose the extra 50 euro cents during this game. The chance of winning an extra 5 euros is 10%. Please click 'Start' to activate the game."

Measure for beliefs in superstition (the same as in Study 1 and Study 2.).

References

1. Grable J, Lytton RH. Financial risk tolerance revisited: the development of a risk assessment instrument. Financial services review. 1999 Jan 1;8(3):163-81.
2. Blais AR, Weber EU. A domain-specific risk-taking (DOSPERT) scale for adult populations. Judgment and Decision making. 2006 Jul 1;1(1).
3. Schwarzer R & Jerusalem M. Generalized Self-Efficacy scale. In J. Weinman, S. Wright, & M. Johnston, Measures in Health Psychology: A User's Portfolio. Causal and Control Beliefs (pp. 35-37). 1995. Windsor, England: NFER-NELSON.
